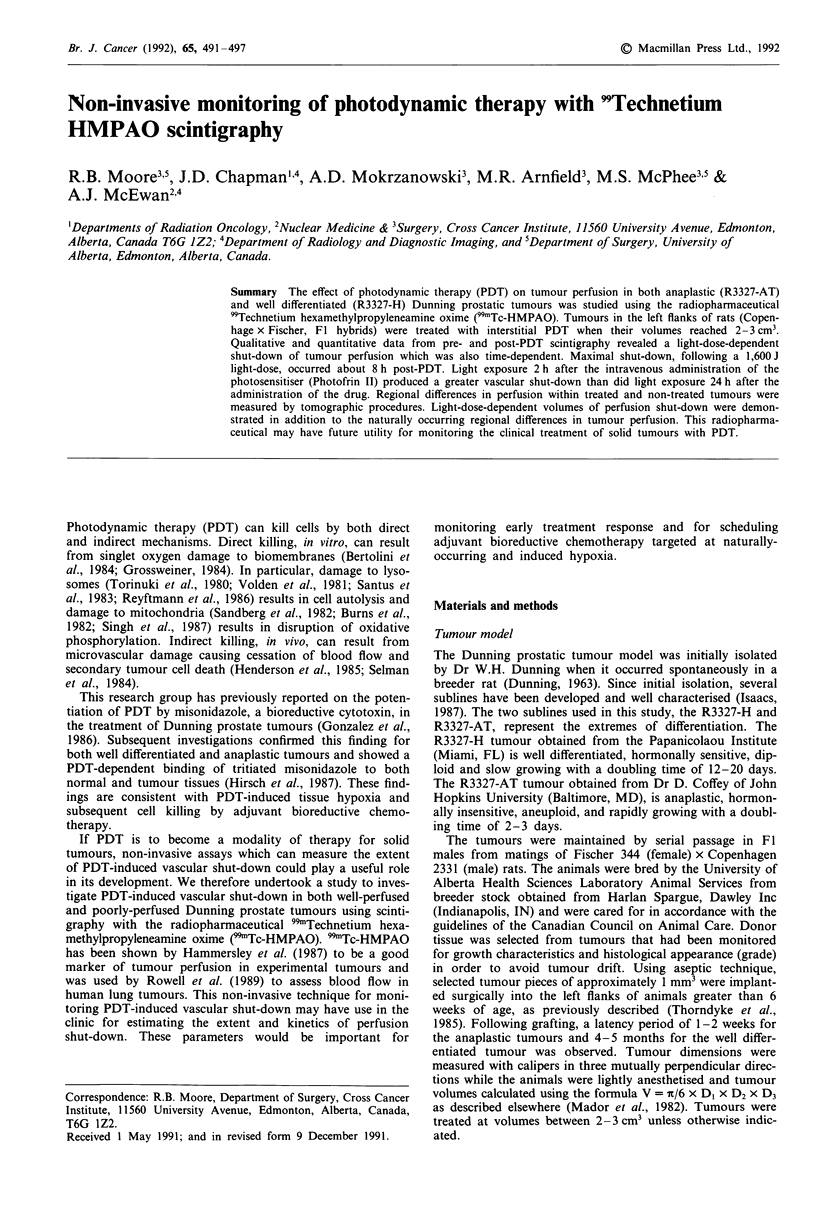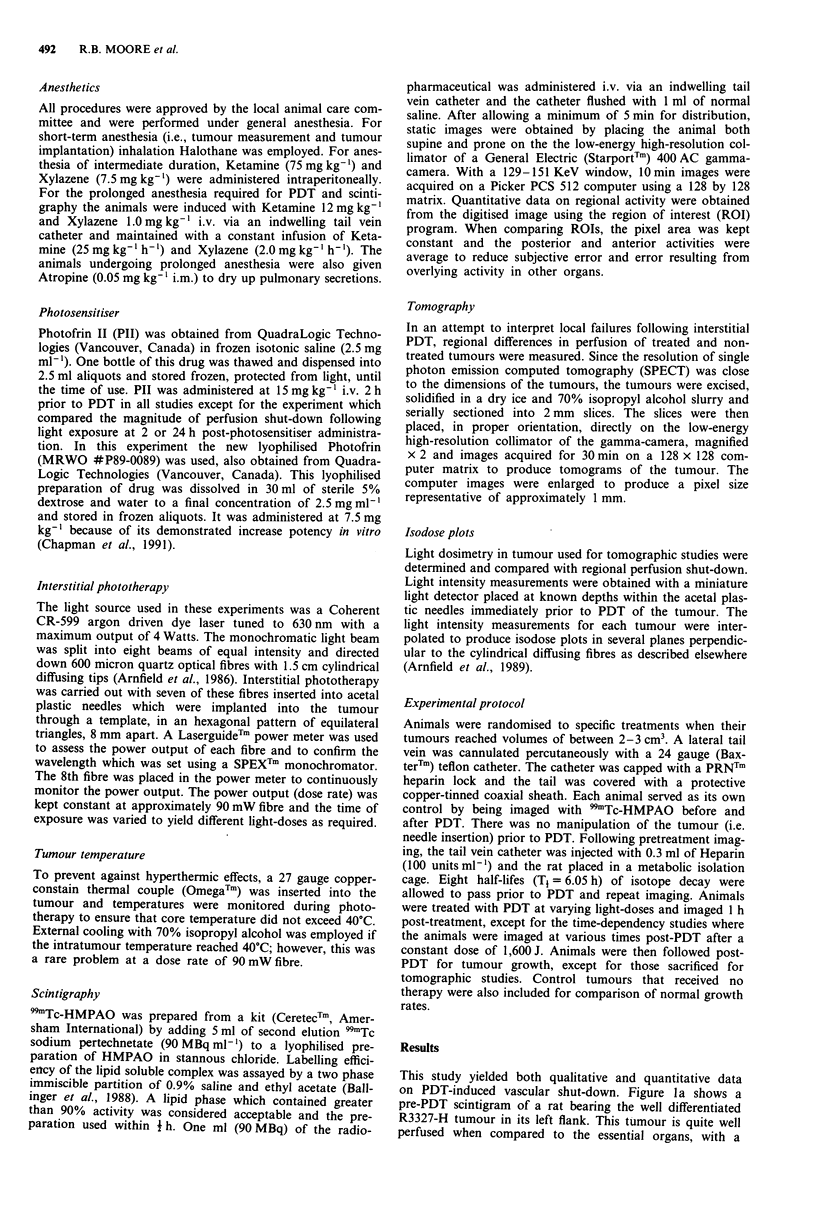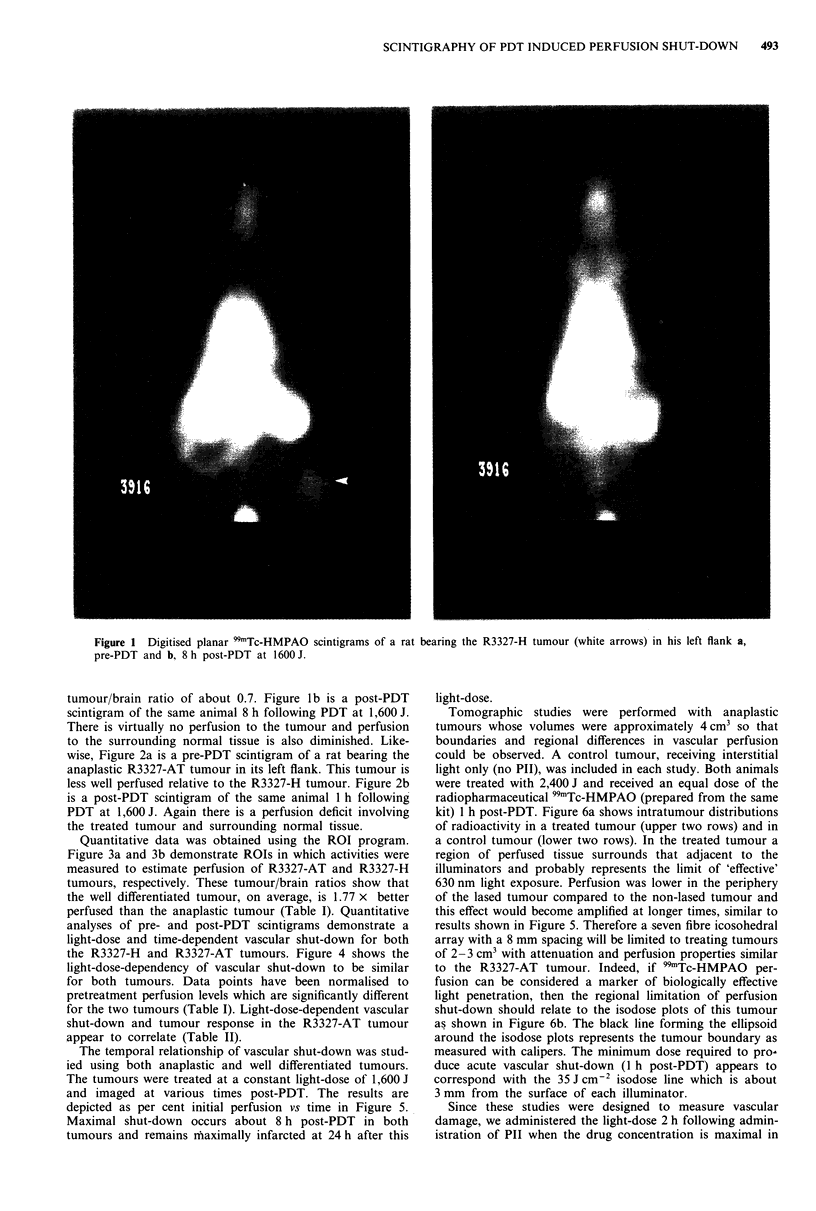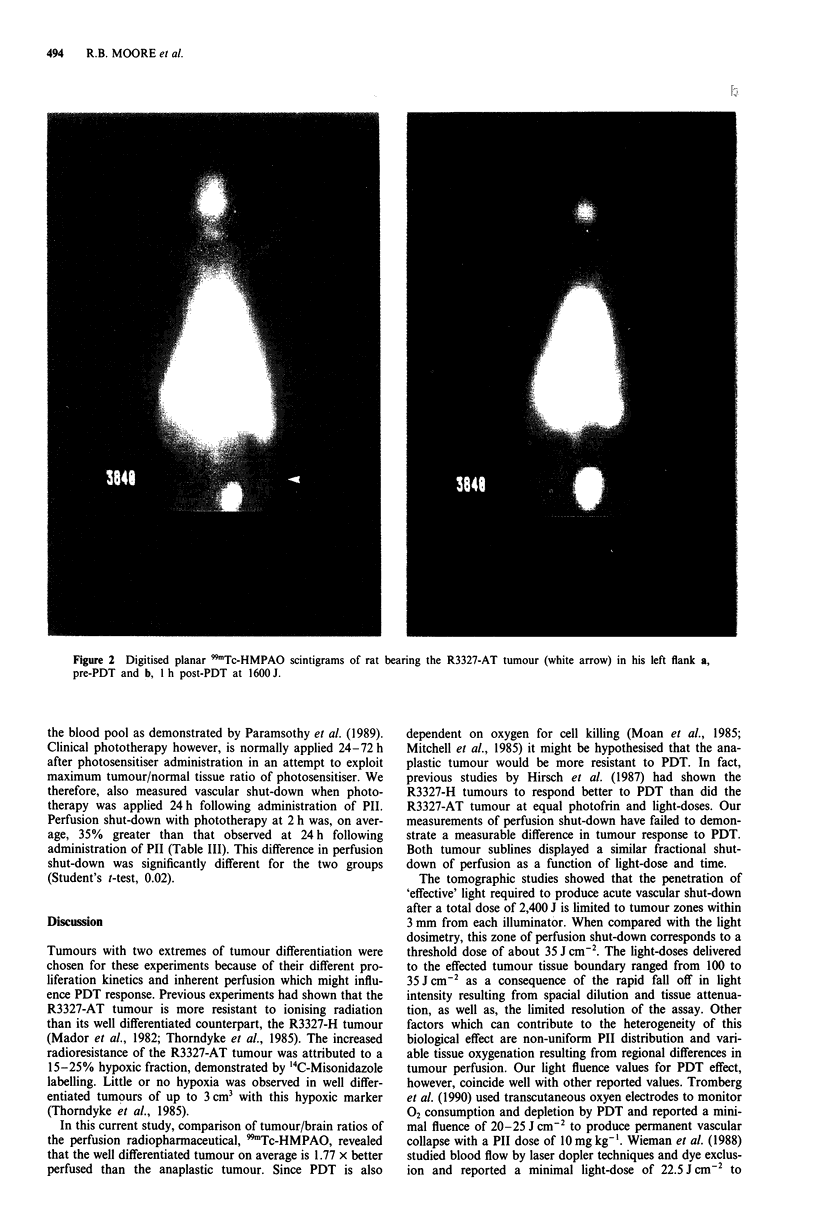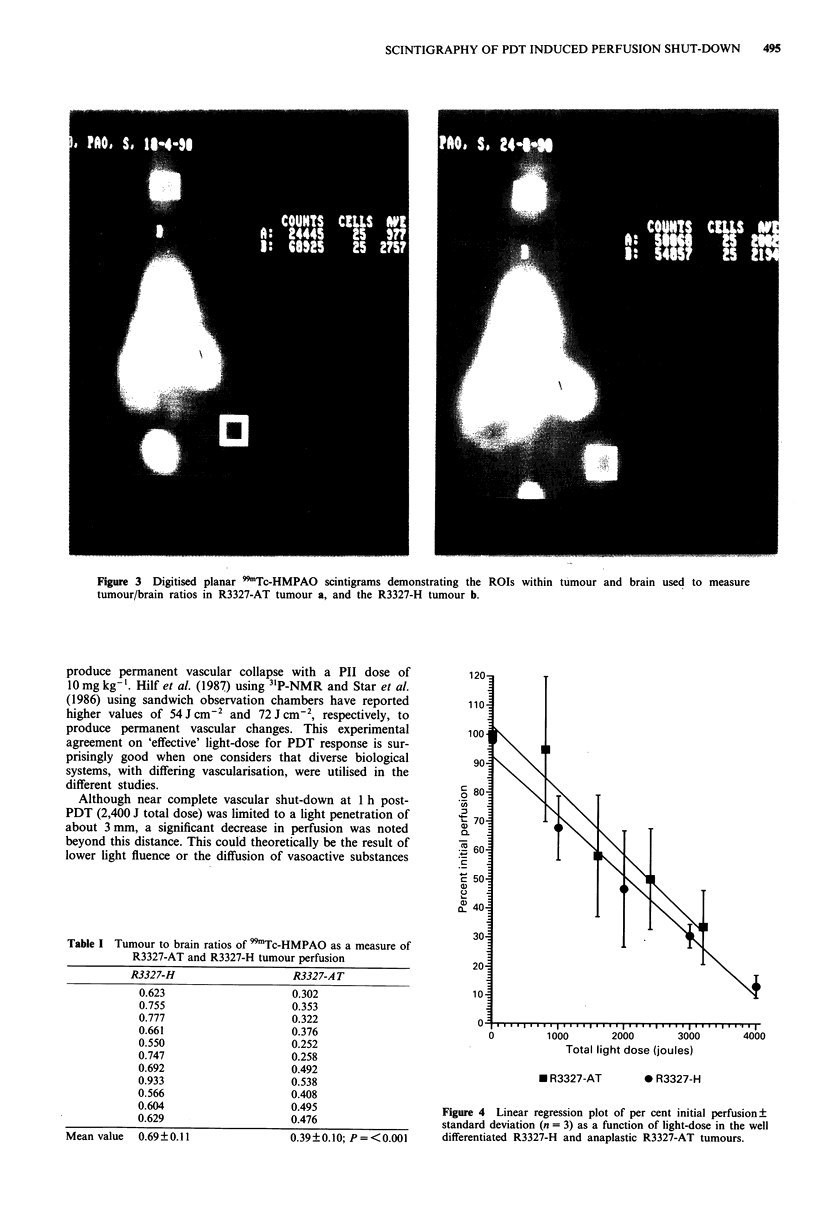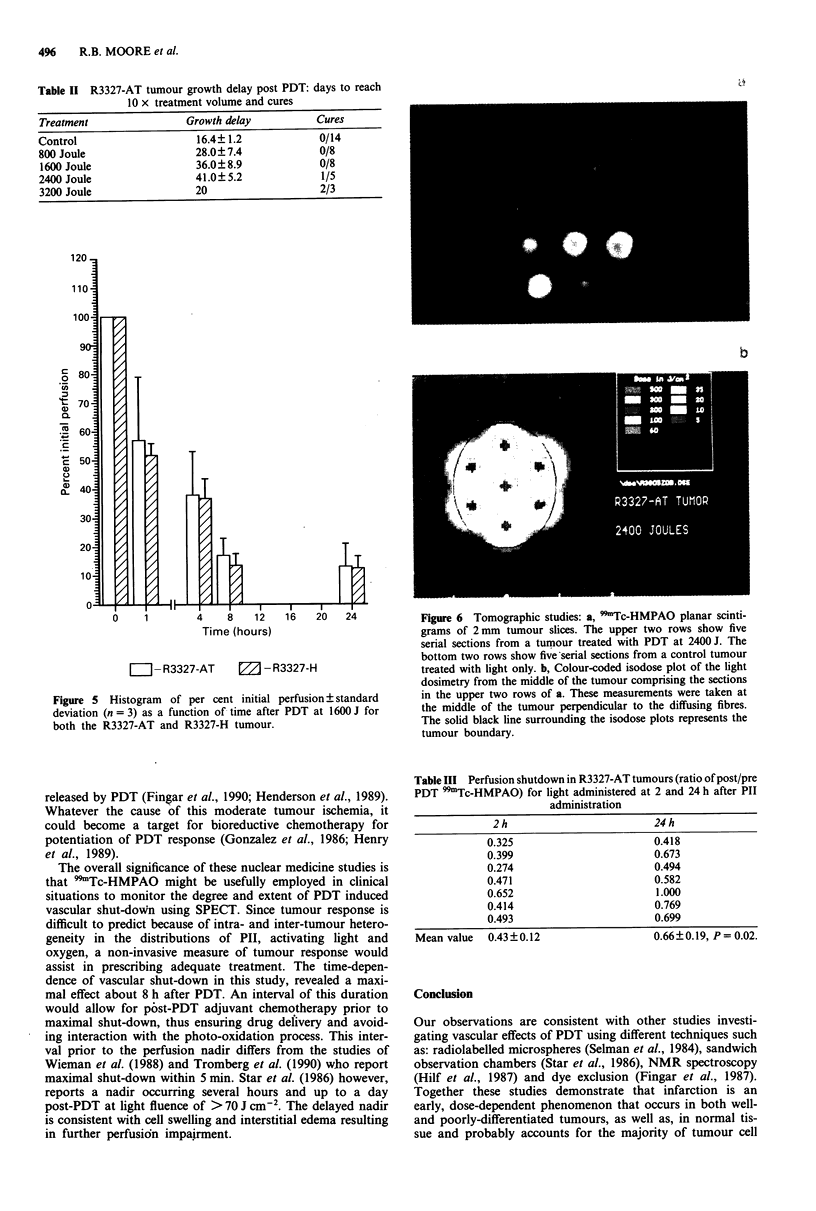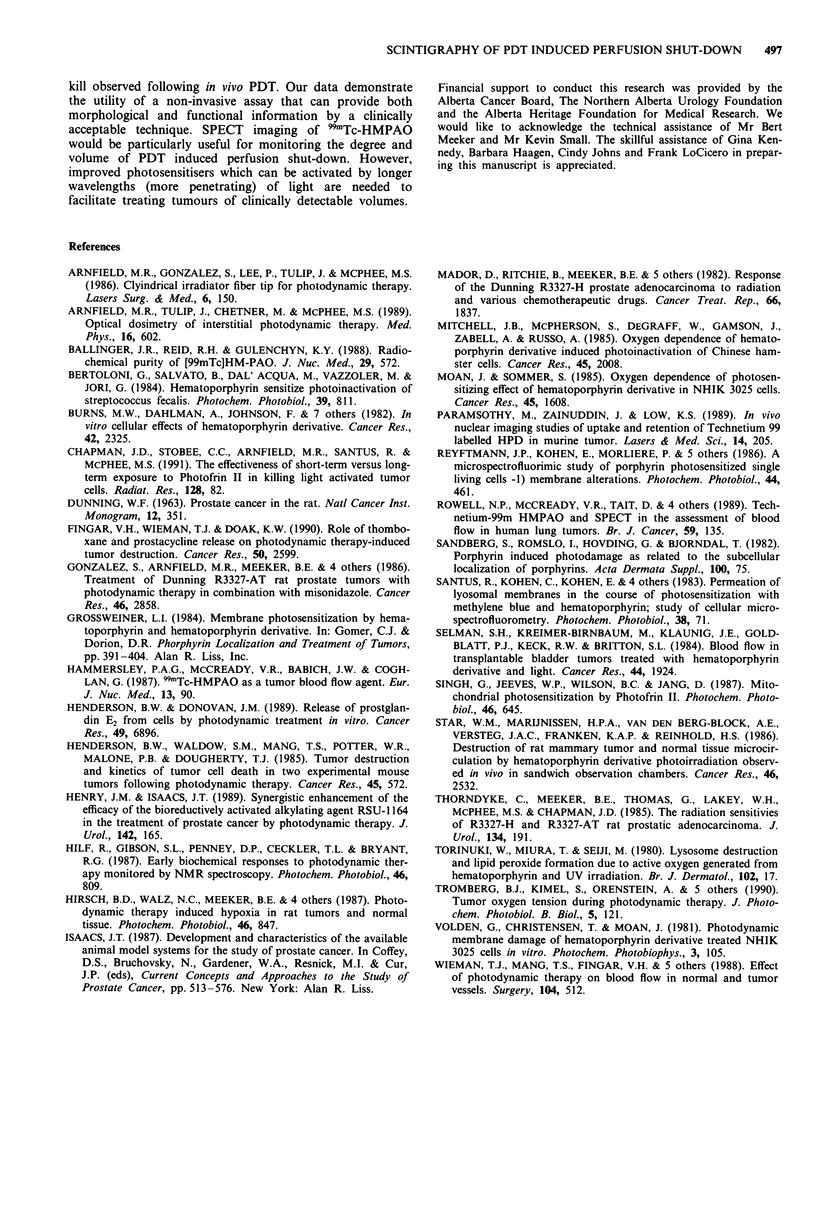# Non-invasive monitoring of photodynamic therapy with 99technetium HMPAO scintigraphy.

**DOI:** 10.1038/bjc.1992.102

**Published:** 1992-04

**Authors:** R. B. Moore, J. D. Chapman, A. D. Mokrzanowski, M. R. Arnfield, M. S. McPhee, A. J. McEwan

**Affiliations:** Department of Radiation Oncology, Cross Cancer Institute, Edmonton, Alberta, Canada.

## Abstract

**Images:**


Br. J. Cancer (1992), 65, 491–497 © Macmillan Press Ltd., 1992

### Non-invasive monitoring of photodynamic therapy with $^{99}$Technetium HMPAO scintigraphy

R.B. Moore[3,5], J.D. Chapman[1,4], A.D. Mokrzanowski[3], M.R. Arnfield[3], M.S. McPhee[3,5] & A.J. McEwan[2,4]

[1]*Departments of Radiation Oncology,* [2]*Nuclear Medicine &* [3]*Surgery, Cross Cancer Institute, 11560 University Avenue, Edmonton, Alberta, Canada T6G 1Z2;* [4]*Department of Radiology and Diagnostic Imaging, and* [5]*Department of Surgery, University of Alberta, Edmonton, Alberta, Canada.*

**Summary** The effect of photodynamic therapy (PDT) on tumour perfusion in both anaplastic (R3327-AT) and well differentiated (R3327-H) Dunning prostatic tumours was studied using the radiopharmaceutical $^{99}$Technetium hexamethylpropyleneamine oxime ($^{99m}$Tc-HMPAO). Tumours in the left flanks of rats (Copenhage × Fischer, F1 hybrids) were treated with interstitial PDT when their volumes reached 2–3 cm$^3$. Qualitative and quantitative data from pre- and post-PDT scintigraphy revealed a light-dose-dependent shut-down of tumour perfusion which was also time-dependent. Maximal shut-down, following a 1,600 J light-dose, occurred about 8 h post-PDT. Light exposure 2 h after the intravenous administration of the photosensitiser (Photofrin II) produced a greater vascular shut-down than did light exposure 24 h after the administration of the drug. Regional differences in perfusion within treated and non-treated tumours were measured by tomographic procedures. Light-dose-dependent volumes of perfusion shut-down were demonstrated in addition to the naturally occurring regional differences in tumour perfusion. This radiopharmaceutical may have future utility for monitoring the clinical treatment of solid tumours with PDT.

Photodynamic therapy (PDT) can kill cells by both direct and indirect mechanisms. Direct killing, *in vitro*, can result from singlet oxygen damage to biomembranes (Bertolini *et al.*, 1984; Grossweiner, 1984). In particular, damage to lysosomes (Torinuki *et al.*, 1980; Volden *et al.*, 1981; Santus *et al.*, 1983; Reyftmann *et al.*, 1986) results in cell autolysis and damage to mitochondria (Sandberg *et al.*, 1982; Burns *et al.*, 1982; Singh *et al.*, 1987) results in disruption of oxidative phosphorylation. Indirect killing, *in vivo*, can result from microvascular damage causing cessation of blood flow and secondary tumour cell death (Henderson *et al.*, 1985; Selman *et al.*, 1984).

This research group has previously reported on the potentiation of PDT by misonidazole, a bioreductive cytotoxin, in the treatment of Dunning prostate tumours (Gonzalez *et al.*, 1986). Subsequent investigations confirmed this finding for both well differentiated and anaplastic tumours and showed a PDT-dependent binding of tritiated misonidazole to both normal and tumour tissues (Hirsch *et al.*, 1987). These findings are consistent with PDT-induced tissue hypoxia and subsequent cell killing by adjuvant bioreductive chemotherapy.

If PDT is to become a modality of therapy for solid tumours, non-invasive assays which can measure the extent of PDT-induced vascular shut-down could play a useful role in its development. We therefore undertook a study to investigate PDT-induced vascular shut-down in both well-perfused and poorly-perfused Dunning prostate tumours using scintigraphy with the radiopharmaceutical $^{99m}$Technetium hexamethylpropyleneamine oxime ($^{99m}$Tc-HMPAO). $^{99m}$Tc-HMPAO has been shown by Hammersley *et al.* (1987) to be a good marker of tumour perfusion in experimental tumours and was used by Rowell *et al.* (1989) to assess blood flow in human lung tumours. This non-invasive technique for monitoring PDT-induced vascular shut-down may have use in the clinic for estimating the extent and kinetics of perfusion shut-down. These parameters would be important for monitoring early treatment response and for scheduling adjuvant bioreductive chemotherapy targeted at naturally-occurring and induced hypoxia.

Correspondence: R.B. Moore, Department of Surgery, Cross Cancer Institute, 11560 University Avenue, Edmonton, Alberta, Canada, T6G 1Z2.
Received 1 May 1991; and in revised form 9 December 1991.

### Materials and methods

### Tumour model

The Dunning prostatic tumour model was initially isolated by Dr W.H. Dunning when it occurred spontaneously in a breeder rat (Dunning, 1963). Since initial isolation, several sublines have been developed and well characterised (Isaacs, 1987). The two sublines used in this study, the R3327-H and R3327-AT, represent the extremes of differentiation. The R3327-H tumour obtained from the Papanicolaou Institute (Miami, FL) is well differentiated, hormonally sensitive, diploid and slow growing with a doubling time of 12–20 days. The R3327-AT tumour obtained from Dr D. Coffey of John Hopkins University (Baltimore, MD), is anaplastic, hormonally insensitive, aneuploid, and rapidly growing with a doubling time of 2–3 days.

The tumours were maintained by serial passage in F1 males from matings of Fischer 344 (female) × Copenhagen 2331 (male) rats. The animals were bred by the University of Alberta Health Sciences Laboratory Animal Services from breeder stock obtained from Harlan Spargue, Dawley Inc (Indianapolis, IN) and were cared for in accordance with the guidelines of the Canadian Council on Animal Care. Donor tissue was selected from tumours that had been monitored for growth characteristics and histological appearance (grade) in order to avoid tumour drift. Using aseptic technique, selected tumour pieces of approximately 1 mm$^3$ were implanted surgically into the left flanks of animals greater than 6 weeks of age, as previously described (Thorndyke *et al.*, 1985). Following grafting, a latency period of 1–2 weeks for the anaplastic tumours and 4–5 months for the well differentiated tumour was observed. Tumour dimensions were measured with calipers in three mutually perpendicular directions while the animals were lightly anesthetised and tumour volumes calculated using the formula $V = \pi/6 \times D_1 \times D_2 \times D_3$ as described elsewhere (Mador *et al.*, 1982). Tumours were treated at volumes between 2–3 cm$^3$ unless otherwise indicated.

*Anesthetics*

All procedures were approved by the local animal care committee and were performed under general anesthesia. For short-term anesthesia (i.e., tumour measurement and tumour implantation) inhalation Halothane was employed. For anesthesia of intermediate duration, Ketamine (75 mg $kg^{-1}$) and Xylazene (7.5 mg $kg^{-1}$) were administered intraperitoneally. For the prolonged anesthesia required for PDT and scintigraphy the animals were induced with Ketamine 12 mg $kg^{-1}$ and Xylazene 1.0 mg $kg^{-1}$ i.v. via an indwelling tail vein catheter and maintained with a constant infusion of Ketamine (25 mg $kg^{-1}$ $h^{-1}$) and Xylazene (2.0 mg $kg^{-1}$ $h^{-1}$). The animals undergoing prolonged anesthesia were also given Atropine (0.05 mg $kg^{-1}$ i.m.) to dry up pulmonary secretions.

*Photosensitiser*

Photofrin II (PII) was obtained from QuadraLogic Technologies (Vancouver, Canada) in frozen isotonic saline (2.5 mg $ml^{-1}$). One bottle of this drug was thawed and dispensed into 2.5 ml aliquots and stored frozen, protected from light, until the time of use. PII was administered at 15 mg $kg^{-1}$ i.v. 2 h prior to PDT in all studies except for the experiment which compared the magnitude of perfusion shut-down following light exposure at 2 or 24 h post-photosensitiser administration. In this experiment the new lyophilised Photofrin (MRWO #P89-0089) was used, also obtained from QuadraLogic Technologies (Vancouver, Canada). This lyophilised preparation of drug was dissolved in 30 ml of sterile 5% dextrose and water to a final concentration of 2.5 mg $ml^{-1}$ and stored in frozen aliquots. It was administered at 7.5 mg $kg^{-1}$ because of its demonstrated increase potency *in vitro* (Chapman *et al.*, 1991).

*Interstitial phototherapy*

The light source used in these experiments was a Coherent CR-599 argon driven dye laser tuned to 630 nm with a maximum output of 4 Watts. The monochromatic light beam was split into eight beams of equal intensity and directed down 600 micron quartz optical fibres with 1.5 cm cylindrical diffusing tips (Arnfield *et al.*, 1986). Interstitial phototherapy was carried out with seven of these fibres inserted into acetal plastic needles which were implanted into the tumour through a template, in an hexagonal pattern of equilateral triangles, 8 mm apart. A Laserguide$^{Tm}$ power meter was used to assess the power output of each fibre and to confirm the wavelength which was set using a SPEX$^{Tm}$ monochromator. The 8th fibre was placed in the power meter to continuously monitor the power output. The power output (dose rate) was kept constant at approximately 90 mW fibre and the time of exposure was varied to yield different light-doses as required.

*Tumour temperature*

To prevent against hyperthermic effects, a 27 gauge copper-constain thermal couple (Omega$^{Tm}$) was inserted into the tumour and temperatures were monitored during phototherapy to ensure that core temperature did not exceed 40°C. External cooling with 70% isopropyl alcohol was employed if the intratumour temperature reached 40°C; however, this was a rare problem at a dose rate of 90 mW fibre.

*Scintigraphy*

$^{99m}$Tc-HMPAO was prepared from a kit (Ceretec$^{Tm}$, Amersham International) by adding 5 ml of second elution $^{99m}$Tc sodium pertechnetate (90 MBq $ml^{-1}$) to a lyophilised preparation of HMPAO in stannous chloride. Labelling efficiency of the lipid soluble complex was assayed by a two phase immiscible partition of 0.9% saline and ethyl acetate (Ballinger *et al.*, 1988). A lipid phase which contained greater than 90% activity was considered acceptable and the preparation used within ½ h. One ml (90 MBq) of the radiopharmaceutical was administered i.v. via an indwelling tail vein catheter and the catheter flushed with 1 ml of normal saline. After allowing a minimum of 5 min for distribution, static images were obtained by placing the animal both supine and prone on the the low-energy high-resolution collimator of a General Electric (Starport$^{Tm}$) 400 AC gamma-camera. With a 129–151 KeV window, 10 min images were acquired on a Picker PCS 512 computer using a 128 by 128 matrix. Quantitative data on regional activity were obtained from the digitised image using the region of interest (ROI) program. When comparing ROIs, the pixel area was kept constant and the posterior and anterior activities were average to reduce subjective error and error resulting from overlying activity in other organs.

*Tomography*

In an attempt to interpret local failures following interstitial PDT, regional differences in perfusion of treated and non-treated tumours were measured. Since the resolution of single photon emission computed tomography (SPECT) was close to the dimensions of the tumours, the tumours were excised, solidified in a dry ice and 70% isopropyl alcohol slurry and serially sectioned into 2 mm slices. The slices were then placed, in proper orientation, directly on the low-energy high-resolution collimator of the gamma-camera, magnified × 2 and images acquired for 30 min on a 128 × 128 computer matrix to produce tomograms of the tumour. The computer images were enlarged to produce a pixel size representative of approximately 1 mm.

*Isodose plots*

Light dosimetry in tumour used for tomographic studies were determined and compared with regional perfusion shut-down. Light intensity measurements were obtained with a miniature light detector placed at known depths within the acetal plastic needles immediately prior to PDT of the tumour. The light intensity measurements for each tumour were interpolated to produce isodose plots in several planes perpendicular to the cylindrical diffusing fibres as described elsewhere (Arnfield *et al.*, 1989).

*Experimental protocol*

Animals were randomised to specific treatments when their tumours reached volumes of between 2–3 $cm^3$. A lateral tail vein was cannulated percutaneously with a 24 gauge (Baxter$^{Tm}$) teflon catheter. The catheter was capped with a PRN$^{Tm}$ heparin lock and the tail was covered with a protective copper-tinned coaxial sheath. Each animal served as its own control by being imaged with $^{99m}$Tc-HMPAO before and after PDT. There was no manipulation of the tumour (i.e. needle insertion) prior to PDT. Following pretreatment imaging, the tail vein catheter was injected with 0.3 ml of Heparin (100 units $ml^{-1}$) and the rat placed in a metabolic isolation cage. Eight half-lifes ($T_{½}$ = 6.05 h) of isotope decay were allowed to pass prior to PDT and repeat imaging. Animals were treated with PDT at varying light-doses and imaged 1 h post-treatment, except for the time-dependency studies where the animals were imaged at various times post-PDT after a constant dose of 1,600 J. Animals were then followed post-PDT for tumour growth, except for those sacrificed for tomographic studies. Control tumours that received no therapy were also included for comparison of normal growth rates.

### Results

This study yielded both qualitative and quantitative data on PDT-induced vascular shut-down. Figure 1a shows a pre-PDT scintigram of a rat bearing the well differentiated R3327-H tumour in its left flank. This tumour is quite well perfused when compared to the essential organs, with a

**Figure 1** Digitised planar $^{99m}$Tc-HMPAO scintigrams of a rat bearing the R3327-H tumour (white arrows) in his left flank **a**, pre-PDT and **b**, 8 h post-PDT at 1600 J.

tumour/brain ratio of about 0.7. Figure 1b is a post-PDT scintigram of the same animal 8 h following PDT at 1,600 J. There is virtually no perfusion to the tumour and perfusion to the surrounding normal tissue is also diminished. Likewise, Figure 2a is a pre-PDT scintigram of a rat bearing the anaplastic R3327-AT tumour in its left flank. This tumour is less well perfused relative to the R3327-H tumour. Figure 2b is a post-PDT scintigram of the same animal 1 h following PDT at 1,600 J. Again there is a perfusion deficit involving the treated tumour and surrounding normal tissue.

Quantitative data was obtained using the ROI program. Figure 3a and 3b demonstrate ROIs in which activities were measured to estimate perfusion of R3327-AT and R3327-H tumours, respectively. These tumour/brain ratios show that the well differentiated tumour, on average, is 1.77 × better perfused than the anaplastic tumour (Table I). Quantitative analyses of pre- and post-PDT scintigrams demonstrate a light-dose and time-dependent vascular shut-down for both the R3327-H and R3327-AT tumours. Figure 4 shows the light-dose-dependency of vascular shut-down to be similar for both tumours. Data points have been normalised to pretreatment perfusion levels which are significantly different for the two tumours (Table I). Light-dose-dependent vascular shut-down and tumour response in the R3327-AT tumour appear to correlate (Table II).

The temporal relationship of vascular shut-down was studied using both anaplastic and well differentiated tumours. The tumours were treated at a constant light-dose of 1,600 J and imaged at various times post-PDT. The results are depicted as per cent initial perfusion *vs* time in Figure 5. Maximal shut-down occurs about 8 h post-PDT in both tumours and remains maximally infarcted at 24 h after this light-dose.

Tomographic studies were performed with anaplastic tumours whose volumes were approximately 4 cm$^3$ so that boundaries and regional differences in vascular perfusion could be observed. A control tumour, receiving interstitial light only (no PII), was included in each study. Both animals were treated with 2,400 J and received an equal dose of the radiopharmaceutical $^{99m}$Tc-HMPAO (prepared from the same kit) 1 h post-PDT. Figure 6a shows intratumour distributions of radioactivity in a treated tumour (upper two rows) and in a control tumour (lower two rows). In the treated tumour a region of perfused tissue surrounds that adjacent to the illuminators and probably represents the limit of 'effective' 630 nm light exposure. Perfusion was lower in the periphery of the lased tumour compared to the non-lased tumour and this effect would become amplified at longer times, similar to results shown in Figure 5. Therefore a seven fibre icosohedral array with a 8 mm spacing will be limited to treating tumours of 2–3 cm$^3$ with attenuation and perfusion properties similar to the R3327-AT tumour. Indeed, if $^{99m}$Tc-HMPAO perfusion can be considered a marker of biologically effective light penetration, then the regional limitation of perfusion shut-down should relate to the isodose plots of this tumour as shown in Figure 6b. The black line forming the ellipsoid around the isodose plots represents the tumour boundary as measured with calipers. The minimum dose required to produce acute vascular shut-down (1 h post-PDT) appears to correspond with the 35 J cm$^{-2}$ isodose line which is about 3 mm from the surface of each illuminator.

Since these studies were designed to measure vascular damage, we administered the light-dose 2 h following administration of PII when the drug concentration is maximal in

**Figure 2** Digitised planar $^{99m}$Tc-HMPAO scintigrams of rat bearing the R3327-AT tumour (white arrow) in his left flank **a**, pre-PDT and **b**, 1 h post-PDT at 1600 J.

the blood pool as demonstrated by Paramsothy *et al.* (1989). Clinical phototherapy however, is normally applied 24–72 h after photosensitiser administration in an attempt to exploit maximum tumour/normal tissue ratio of photosensitiser. We therefore, also measured vascular shut-down when phototherapy was applied 24 h following administration of PII. Perfusion shut-down with phototherapy at 2 h was, on average, 35% greater than that observed at 24 h following administration of PII (Table III). This difference in perfusion shut-down was significantly different for the two groups (Student's *t*-test, 0.02).

### Discussion

Tumours with two extremes of tumour differentiation were chosen for these experiments because of their different proliferation kinetics and inherent perfusion which might influence PDT response. Previous experiments had shown that the R3327-AT tumour is more resistant to ionising radiation than its well differentiated counterpart, the R3327-H tumour (Mador *et al.*, 1982; Thorndyke *et al.*, 1985). The increased radioresistance of the R3327-AT tumour was attributed to a 15–25% hypoxic fraction, demonstrated by $^{14}$C-Misonidazole labelling. Little or no hypoxia was observed in well differentiated tumours of up to 3 $cm^3$ with this hypoxic marker (Thorndyke *et al.*, 1985).

In this current study, comparison of tumour/brain ratios of the perfusion radiopharmaceutical, $^{99m}$Tc-HMPAO, revealed that the well differentiated tumour on average is 1.77 × better perfused than the anaplastic tumour. Since PDT is also dependent on oxygen for cell killing (Moan *et al.*, 1985; Mitchell *et al.*, 1985) it might be hypothesised that the anaplastic tumour would be more resistant to PDT. In fact, previous studies by Hirsch *et al.* (1987) had shown the R3327-H tumours to respond better to PDT than did the R3327-AT tumour at equal photofrin and light-doses. Our measurements of perfusion shut-down have failed to demonstrate a measurable difference in tumour response to PDT. Both tumour sublines displayed a similar fractional shut-down of perfusion as a function of light-dose and time.

The tomographic studies showed that the penetration of 'effective' light required to produce acute vascular shut-down after a total dose of 2,400 J is limited to tumour zones within 3 mm from each illuminator. When compared with the light dosimetry, this zone of perfusion shut-down corresponds to a threshold dose of about 35 J $cm^{-2}$. The light-doses delivered to the effected tumour tissue boundary ranged from 100 to 35 J $cm^{-2}$ as a consequence of the rapid fall off in light intensity resulting from spacial dilution and tissue attenuation, as well as, the limited resolution of the assay. Other factors which can contribute to the heterogeneity of this biological effect are non-uniform PII distribution and variable tissue oxygenation resulting from regional differences in tumour perfusion. Our light fluence values for PDT effect, however, coincide well with other reported values. Tromberg *et al.* (1990) used transcutaneous oxyen electrodes to monitor $O_2$ consumption and depletion by PDT and reported a minimal fluence of 20–25 J $cm^{-2}$ to produce permanent vascular collapse with a PII dose of 10 mg $kg^{-1}$. Wieman *et al.* (1988) studied blood flow by laser dopler techniques and dye exclusion and reported a minimal light-dose of 22.5 J $cm^{-2}$ to

**Figure 3** Digitised planar $^{99m}$Tc-HMPAO scintigrams demonstrating the ROIs within tumour and brain used to measure tumour/brain ratios in R3327-AT tumour **a**, and the R3327-H tumour **b**.

produce permanent vascular collapse with a PII dose of 10 mg $kg^{-1}$. Hilf *et al.* (1987) using $^{31}$P-NMR and Star *et al.* (1986) using sandwich observation chambers have reported higher values of 54 J $cm^{-2}$ and 72 J $cm^{-2}$, respectively, to produce permanent vascular changes. This experimental agreement on 'effective' light-dose for PDT response is surprisingly good when one considers that diverse biological systems, with differing vascularisation, were utilised in the different studies.

Although near complete vascular shut-down at 1 h post-PDT (2,400 J total dose) was limited to a light penetration of about 3 mm, a significant decrease in perfusion was noted beyond this distance. This could theoretically be the result of lower light fluence or the diffusion of vasoactive substances

**Table I** Tumour to brain ratios of $^{99m}$Tc-HMPAO as a measure of R3327-AT and R3327-H tumour perfusion

| | *R3327-H* | *R3327-AT* |
|---|---|---|
| | 0.623 | 0.302 |
| | 0.755 | 0.353 |
| | 0.777 | 0.322 |
| | 0.661 | 0.376 |
| | 0.550 | 0.252 |
| | 0.747 | 0.258 |
| | 0.692 | 0.492 |
| | 0.933 | 0.538 |
| | 0.566 | 0.408 |
| | 0.604 | 0.495 |
| | 0.629 | 0.476 |
| Mean value | 0.69±0.11 | 0.39±0.10; $P = <0.001$ |

**Figure 4** Linear regression plot of per cent initial perfusion ± standard deviation ($n = 3$) as a function of light-dose in the well differentiated R3327-H and anaplastic R3327-AT tumours.

**Table II** R3327-AT tumour growth delay post PDT: days to reach 10 × treatment volume and cures

| *Treatment* | *Growth delay* | *Cures* |
|---|---|---|
| Control | 16.4±1.2 | 0/14 |
| 800 Joule | 28.0±7.4 | 0/8 |
| 1600 Joule | 36.0±8.9 | 0/8 |
| 2400 Joule | 41.0±5.2 | 1/5 |
| 3200 Joule | 20 | 2/3 |

**Figure 5** Histogram of per cent initial perfusion ± standard deviation ($n = 3$) as a function of time after PDT at 1600 J for both the R3327-AT and R3327-H tumour.

**Figure 6** Tomographic studies: **a**, $^{99m}$Tc-HMPAO planar scintigrams of 2 mm tumour slices. The upper two rows show five serial sections from a tumour treated with PDT at 2400 J. The bottom two rows show five serial sections from a control tumour treated with light only. **b**, Colour-coded isodose plot of the light dosimetry from the middle of the tumour comprising the sections in the upper two rows of **a**. These measurements were taken at the middle of the tumour perpendicular to the diffusing fibres. The solid black line surrounding the isodose plots represents the tumour boundary.

released by PDT (Fingar *et al.*, 1990; Henderson *et al.*, 1989). Whatever the cause of this moderate tumour ischemia, it could become a target for bioreductive chemotherapy for potentiation of PDT response (Gonzalez *et al.*, 1986; Henry *et al.*, 1989).

The overall significance of these nuclear medicine studies is that $^{99m}$Tc-HMPAO might be usefully employed in clinical situations to monitor the degree and extent of PDT induced vascular shut-down using SPECT. Since tumour response is difficult to predict because of intra- and inter-tumour heterogeneity in the distributions of PII, activating light and oxygen, a non-invasive measure of tumour response would assist in prescribing adequate treatment. The time-dependence of vascular shut-down in this study, revealed a maximal effect about 8 h after PDT. An interval of this duration would allow for post-PDT adjuvant chemotherapy prior to maximal shut-down, thus ensuring drug delivery and avoiding interaction with the photo-oxidation process. This interval prior to the perfusion nadir differs from the studies of Wieman *et al.* (1988) and Tromberg *et al.* (1990) who report maximal shut-down within 5 min. Star *et al.* (1986) however, reports a nadir occurring several hours and up to a day post-PDT at light fluence of $>70\ \mathrm{J\ cm^{-2}}$. The delayed nadir is consistent with cell swelling and interstitial edema resulting in further perfusion impairment.

**Table III** Perfusion shutdown in R3327-AT tumours (ratio of post/pre PDT $^{99m}$Tc-HMPAO) for light administered at 2 and 24 h after PII administration

| | *2 h* | *24 h* |
|---|---|---|
| | 0.325 | 0.418 |
| | 0.399 | 0.673 |
| | 0.274 | 0.494 |
| | 0.471 | 0.582 |
| | 0.652 | 1.000 |
| | 0.414 | 0.769 |
| | 0.493 | 0.699 |
| Mean value | 0.43±0.12 | 0.66±0.19, $P = 0.02$. |

### Conclusion

Our observations are consistent with other studies investigating vascular effects of PDT using different techniques such as: radiolabelled microspheres (Selman *et al.*, 1984), sandwich observation chambers (Star *et al.*, 1986), NMR spectroscopy (Hilf *et al.*, 1987) and dye exclusion (Fingar *et al.*, 1987). Together these studies demonstrate that infarction is an early, dose-dependent phenomenon that occurs in both well- and poorly-differentiated tumours, as well as, in normal tissue and probably accounts for the majority of tumour cell kill observed following *in vivo* PDT. Our data demonstrate the utility of a non-invasive assay that can provide both morphological and functional information by a clinically acceptable technique. SPECT imaging of $^{99m}$Tc-HMPAO would be particularly useful for monitoring the degree and volume of PDT induced perfusion shut-down. However, improved photosensitisers which can be activated by longer wavelengths (more penetrating) of light are needed to facilitate treating tumours of clinically detectable volumes.

Financial support to conduct this research was provided by the Alberta Cancer Board, The Northern Alberta Urology Foundation and the Alberta Heritage Foundation for Medical Research. We would like to acknowledge the technical assistance of Mr Bert Meeker and Mr Kevin Small. The skillful assistance of Gina Kennedy, Barbara Haagen, Cindy Johns and Frank LoCicero in preparing this manuscript is appreciated.

### References

ARNFIELD, M.R., GONZALEZ, S., LEE, P., TULIP, J. & MCPHEE, M.S. (1986). Clyindrical irradiator fiber tip for photodynamic therapy. *Lasers Surg. & Med.*, **6**, 150.

ARNFIELD, M.R., TULIP, J., CHETNER, M. & MCPHEE, M.S. (1989). Optical dosimetry of interstitial photodynamic therapy. *Med. Phys.*, **16**, 602.

BALLINGER, J.R., REID, R.H. & GULENCHYN, K.Y. (1988). Radiochemical purity of [99mTc]HM-PAO. *J. Nuc. Med.*, **29**, 572.

BERTOLONI, G., SALVATO, B., DAL' ACQUA, M., VAZZOLER, M. & JORI, G. (1984). Hematoporphyrin sensitize photoinactivation of streptococcus fecalis. *Photochem. Photobiol.*, **39**, 811.

BURNS, M.W., DAHLMAN, A., JOHNSON, F. & 7 others (1982). *In vitro* cellular effects of hematoporphyrin derivative. *Cancer Res.*, **42**, 2325.

CHAPMAN, J.D., STOBEE, C.C., ARNFIELD, M.R., SANTUS, R. & MCPHEE, M.S. (1991). The effectiveness of short-term versus long-term exposure to Photofrin II in killing light activated tumor cells. *Radiat. Res.*, **128**, 82.

DUNNING, W.F. (1963). Prostate cancer in the rat. *Natl Cancer Inst. Monogram*, **12**, 351.

FINGAR, V.H., WIEMAN, T.J. & DOAK, K.W. (1990). Role of thombo-xane and prostacycline release on photodynamic therapy-induced tumor destruction. *Cancer Res.*, **50**, 2599.

GONZALEZ, S., ARNFIELD, M.R., MEEKER, B.E. & 4 others (1986). Treatment of Dunning R3327-AT rat prostate tumors with photodynamic therapy in combination with misonidazole. *Cancer Res.*, **46**, 2858.

GROSSWEINER, L.I. (1984). Membrane photosensitization by hematoporphyrin and hematoporphyrin derivative. In: Gomer, C.J. & Dorion, D.R. *Phorphyrin Localization and Treatment of Tumors*, pp. 391–404. Alan R. Liss, Inc.

HAMMERSLEY, P.A.G., MCCREADY, V.R., BABICH, J.W. & COGHLAN, G. (1987). $^{99m}$Tc-HMPAO as a tumor blood flow agent. *Eur. J. Nuc. Med.*, **13**, 90.

HENDERSON, B.W. & DONOVAN, J.M. (1989). Release of prostglandin $E_2$ from cells by photodynamic treatment *in vitro*. *Cancer Res.*, **49**, 6896.

HENDERSON, B.W., WALDOW, S.M., MANG, T.S., POTTER, W.R., MALONE, P.B. & DOUGHERTY, T.J. (1985). Tumor destruction and kinetics of tumor cell death in two experimental mouse tumors following photodynamic therapy. *Cancer Res.*, **45**, 572.

HENRY, J.M. & ISAACS, J.T. (1989). Synergistic enhancement of the efficacy of the bioreductively activated alkylating agent RSU-1164 in the treatment of prostate cancer by photodynamic therapy. *J. Urol.*, **142**, 165.

HILF, R., GIBSON, S.L., PENNEY, D.P., CECKLER, T.L. & BRYANT, R.G. (1987). Early biochemical responses to photodynamic therapy monitored by NMR spectroscopy. *Photochem. Photobiol.*, **46**, 809.

HIRSCH, B.D., WALZ, N.C., MEEKER, B.E. & 4 others (1987). Photodynamic therapy induced hypoxia in rat tumors and normal tissue. *Photochem. Photobiol.*, **46**, 847.

ISAACS, J.T. (1987). Development and characteristics of the available animal model systems for the study of prostate cancer. In Coffey, D.S., Bruchovsky, N., Gardener, W.A., Resnick, M.I. & Cur, J.P. (eds), *Current Concepts and Approaches to the Study of Prostate Cancer*, pp. 513–576. New York: Alan R. Liss.

MADOR, D., RITCHIE, B., MEEKER, B.E. & 5 others (1982). Response of the Dunning R3327-H prostate adenocarcinoma to radiation and various chemotherapeutic drugs. *Cancer Treat. Rep.*, **66**, 1837.

MITCHELL, J.B., MCPHERSON, S., DEGRAFF, W., GAMSON, J., ZABELL, A. & RUSSO, A. (1985). Oxygen dependence of hematoporphyrin derivative induced photoinactivation of Chinese hamster cells. *Cancer Res.*, **45**, 2008.

MOAN, J. & SOMMER, S. (1985). Oxygen dependence of photosensitizing effect of hematoporphyrin derivative in NHIK 3025 cells. *Cancer Res.*, **45**, 1608.

PARAMSOTHY, M., ZAINUDDIN, J. & LOW, K.S. (1989). *In vivo* nuclear imaging studies of uptake and retention of Technetium 99 labelled HPD in murine tumor. *Lasers & Med. Sci.*, **14**, 205.

REYFTMANN, J.P., KOHEN, E., MORLIERE, P. & 5 others (1986). A microspectrofluorimic study of porphyrin photosensitized single living cells -1) membrane alterations. *Photochem. Photobiol.*, **44**, 461.

ROWELL, N.P., MCCREADY, V.R., TAIT, D. & 4 others (1989). Technetium-99m HMPAO and SPECT in the assessment of blood flow in human lung tumors. *Br. J. Cancer*, **59**, 135.

SANDBERG, S., ROMSLO, I., HOVDING, G. & BJORNDAL, T. (1982). Porphyrin induced photodamage as related to the subcellular localization of porphyrins. *Acta Dermata Suppl.*, **100**, 75.

SANTUS, R., KOHEN, C., KOHEN, E. & 4 others (1983). Permeation of lyosomal membranes in the course of photosensitization with methylene blue and hematoporphyrin; study of cellular microspectrofluorometry. *Photochem. Photobiol.*, **38**, 71.

SELMAN, S.H., KREIMER-BIRNBAUM, M., KLAUNIG, J.E., GOLDBLATT, P.J., KECK, R.W. & BRITTON, S.L. (1984). Blood flow in transplantable bladder tumors treated with hematoporphyrin derivative and light. *Cancer Res.*, **44**, 1924.

SINGH, G., JEEVES, W.P., WILSON, B.C. & JANG, D. (1987). Mitochondrial photosensitization by Photofrin II. *Photochem. Photobiol.*, **46**, 645.

STAR, W.M., MARIJNISSEN, H.P.A., VAN DEN BERG-BLOCK, A.E., VERSTEG, J.A.C., FRANKEN, K.A.P. & REINHOLD, H.S. (1986). Destruction of rat mammary tumor and normal tissue microcirculation by hematoporphyrin derivative photoirradiation observed *in vivo* in sandwich observation chambers. *Cancer Res.*, **46**, 2532.

THORNDYKE, C., MEEKER, B.E., THOMAS, G., LAKEY, W.H., MCPHEE, M.S. & CHAPMAN, J.D. (1985). The radiation sensitivies of R3327-H and R3327-AT rat prostatic adenocarcinoma. *J. Urol.*, **134**, 191.

TORINUKI, W., MIURA, T. & SEIJI, M. (1980). Lysosome destruction and lipid peroxide formation due to active oxygen generated from hematoporphyrin and UV irradiation. *Br. J. Dermatol.*, **102**, 17.

TROMBERG, B.J., KIMEL, S., ORENSTEIN, A. & 5 others (1990). Tumor oxygen tension during photodynamic therapy. *J. Photochem. Photobiol. B. Biol.*, **5**, 121.

VOLDEN, G., CHRISTENSEN, T. & MOAN, J. (1981). Photodynamic membrane damage of hematoporphyrin derivative treated NHIK 3025 cells *in vitro*. *Photochem. Photobiophys.*, **3**, 105.

WIEMAN, T.J., MANG, T.S., FINGAR, V.H. & 5 others (1988). Effect of photodynamic therapy on blood flow in normal and tumor vessels. *Surgery*, **104**, 512.